# Acute hemorrhagic leukoencephalitis with gradual symptom onset: Case Report and literature review

**DOI:** 10.3389/fnins.2025.1557046

**Published:** 2025-03-31

**Authors:** Ida Sannes, Marianne E. Harr, Ellen-Ann Antal, Mona K. Beyer, Kaja Nordengen

**Affiliations:** ^1^Faculty of Medicine, Institute of Clinical Medicine, University of Oslo, Oslo, Norway; ^2^Department of Neurosurgery, Oslo University Hospital, Oslo, Norway; ^3^Department of Pathology, Oslo University Hospital, Oslo, Norway; ^4^Division of Radiology and Nuclear Medicine, Oslo University Hospital, Oslo, Norway; ^5^Department of Neurology, Oslo University Hospital, Oslo, Norway

**Keywords:** case report, AHLE, neuroinflammation, neuropathology, Hurst syndrome

## Abstract

This report highlights an atypical case of acute hemorrhagic Leukoencephalitis (AHLE) in a 73-year-old male, contributing valuable insights into the disease’s progression in older adults. AHLE, a rare and often fatal central nervous system disorder typically affecting younger individuals, with a median age of 33. Our patient experienced atypical subacute symptoms over 3 months, the longest duration reported, and ultimately achieved a functional outcome with a modified Rankin Scale (mRS) score of 2. A comprehensive review of 152 cases available through PubMed, revealing a 58% mortality rate with a median survival of just 2 days, and a mean mRS of 4.3, though survivor exhibited a more favorable mean mRS of 1.8. Only 6% of cases had a subacute onset of 3 weeks or more.

## Introduction

Acute hemorrhagic leukoencephalitis (AHLE), is a rare and severe inflammatory disease of the central nervous system (CNS), that was first described by Hurst (1941). Later, a total of 152 cases have been described, with more than one third of the cases described since 2020. Considered a less common variant of acute disseminated encephalomyelitis (ADEM), AHLE predominately affects young adults, distinguishing it from ADEM, which is mostly observed in pediatric patients. Furthermore, AHLE is often more severe than ADEM, marked by rapid neurological deterioration, with death commonly occurring within the first week of the disease. With a mortality rate at 46.5–70% (Duggal et al., 2014; Grzonka et al., 2020), early recognition is paramount to increase the chances of survival.

The diagnosis relies primarily on clinical assessment, marked by the rapid onset of neurological symptoms, commonly including headache, focal neurological deficits, seizures, hemiparesis, and impaired consciousness. Cerebrospinal fluid (CSF) analysis and magnetic resonance imaging (MRI) provide essential diagnostic support, with MRI typically revealing multifocal white matter lesions with edema, mass effect, and petechial hemorrhages (Grzonka et al., 2020). While the exact etiology remains unclear, AHLE is widely considered a post-infectious autoimmune process, often following a viral upper respiratory tract infection (Alromaihi, 2022).

To date, no established guidelines exist for the treatment of AHLE, but earlier reported successful interventions include high-dose steroids (Cisse et al., 2011), immunoglobulins (Khademi and Aelami, 2016), plasmapheresis (Catalan et al., 2009), or other immunomodulators (Garcia-Castellon et al., 2023). Additionally, certain cases necessitate a decompressive craniectomy to alleviate increased intracranial pressure (Ryan et al., 2007). Despite intervention, mortality rates remain high, and survivors often experience significant neurologic morbidity (Alromaihi, 2022).

In this paper, we present a case of AHLE with classic findings on the diagnostic workups, but with an atypical, subacute course, a rarity in the literature. At the age of 73, the patient also represents one of the oldest reported cases with AHLE. Despite delayed treatment initiation, the patient survived with minor neurological deficits. Additionally, we perform a comprehensive literature review on all published cases focusing on outcome and time to diagnosis (see Supplementary information for search strategy and summary table). This approach seeks to contribute to the understanding and recognition of this rare and severe neurological disorder.

## Case description

A 73-year-old man was admitted to a medical care center following a right-sided dislocated pertrochanteric fracture sustained during a fall. Over the preceding 3 months, the patient had experienced a gradual decline in function, beginning with shuffling gait, memory impairment, behavioral changes, increasingly impaired language comprehension, and decreased fluency of speech. One month prior to admission he had developed global aphasia. Despite a referral to a neurologist for these symptoms, he had not yet secured an appointment.

Upon admission, the patient was awake but somnolent, with a GCS score of 13 (E4, V3, M6), exhibiting pronounced aphasia and dysarthria. The neurological exam revealed reduced muscle strength in the right arm, and slower, less precise movements in the right hand, along with right-sided reflex dominance. The fracture prevented examination of the lower limb.

**Figure 1:**
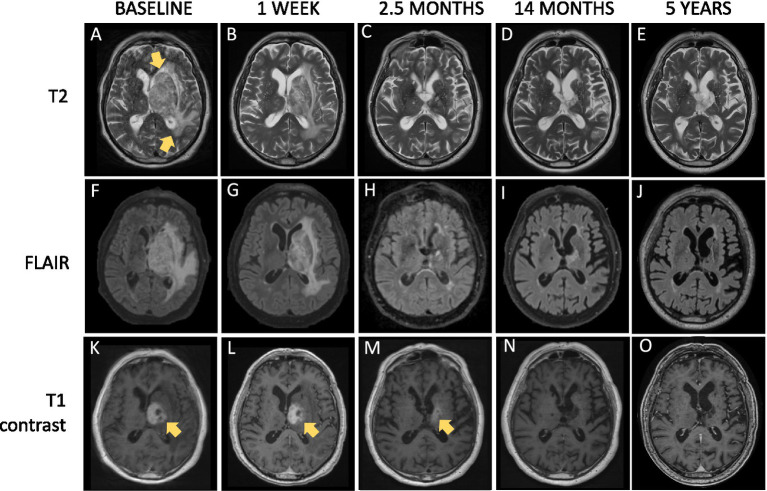
Magnetic resonance imaging (MRI) sequences including T2-weighted **(A–E)**, fluid-attenuated inversion recovery (FLAIR) **(F–J)**, and contrast-enhanced T1-weighted **(K–O)** imaging showcasing the acute phase and follow-up over 5 years. At baseline, a contrast-enhancing tumor measuring 3.3 × 3 × 3.5 cm **(K)** with central necrosis and blood products was observed in the basal ganglia, resulting in compression of the left lateral ventricle. There were marked peritumoral hyperintense changes on T2 **(A)** and FLAIR **(F)** extending to the midbrain, pons, through the left cerebellar peduncle to the left cerebellar hemisphere, as well as the entire left frontal lobe, parietal lobe, and part of the left temporal lobe. In **(A)**, yellow arrows indicate the extent of the lesion and edema on T2 at baseline. Yellow arrows are also used to highlight the central contrast enhancement within the lesion in **(K,L,M)**.

**Figure 2:**
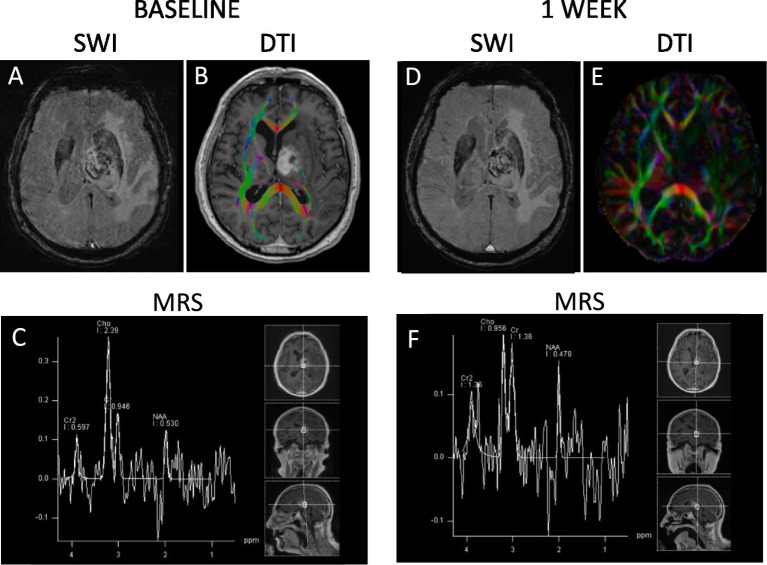
Baseline and one-week follow-up imaging sequences highlighting the progression of the case. **(A,D)** Susceptibility Weighted Imaging (SWI), which emphasizes magnetic susceptibility differences, is shown at baseline **(A)** and after 1 week **(D)**. **(B,E)** Diffusion Tensor Imaging (DTI), used to assess the integrity of neural tracts, is presented at baseline **(B)** and after 1 week **(E)**. **(C,F)** Magnetic Resonance Spectroscopy (MRS), which provides metabolic information of the tissue, is demonstrated at baseline **(C)** and after 1 week **(F)**. SWI indicated susceptibility effects in relation to the contrast-enhancing part of the tumor, as well as small foci in the perirolandic and peritrigonal regions on the right side. DTI showed disruption of nerve tracts, which was interpreted as infiltration. Multivoxel spectroscopy with intermediate echo time revealed pathological spectra in the contrast-enhancing part of the tumor, with a choline (Cho) to N-acetyl-aspartate (NAA) ratio of 4.5. After 1 week, the signal void consistent with hemorrhage remained unchanged. However, there was reduced diffusion signal within the tumor, suggesting a regression in the number of tumor cells. The choline/NAA ratio decreased to 2.0.

A computer tomography (CT) scan revealed a lesion in the left hemisphere with significant perifocal edema pressing against the brainstem, causing mass effect. Brain magnetic resonance imaging (MRI) revealed an expansive tumor deep in the central left hemisphere, with hemorrhagic components, low native T1-signal, increased T2-signal (Figure 1), and enhanced cell density on diffusion-weighted imaging (DWI). The affected area included large parts of the basal ganglia, pars of the mesencephalon, and the left middle cerebellar peduncle. Perifocal edema extended into the frontal lobe, parietal lobe, and parts of the left temporal lobe, causing a notable mass effect. Susceptibility weighted images (SWI) demonstrated blood products in the tumor, while diffusion tensor imaging (DTI) showed destruction of nerve pathways, initially interpreted as infiltration. Multivoxel spectroscopy shows pathological spectra in the contrast-enhancing part of the tumor with a Cho/NAA ratio of 4.5, and a less elevated Cho/NAA ratio around the tumor, within the normal range, see Figure 2. Although both glioblastoma and lymphoma were considered as the two most likely diagnoses initially, the MRI results were atypical for both. Subsequent CT scans of the abdomen, chest, and pelvis did not show signs of primary tumors located in these areas.

After 1 week of steroid treatment, both the contrast enhancement (L) and peritumoral edema showed regression (B and G). The tumor continued to shrink, measuring 1 × 1 cm at 2.5 months (M), located at the level of the internal capsule with central resolution. A subtle enhancement was noted directly posterior for the tumor. By 14 months, only minimal residual enhancement remained in the left basal ganglia region (N), with retraction and Wallerian degeneration on the same side (D, I, and N).

At five years, residual deposition of blood products is still visible deep within the left cerebral hemisphere, affecting the anterior and medial parts of the left thalamus, parts of the globus pallidus, and the periventricular white matter. There is noted ex vacuo retraction of the left lateral ventricle’s cella media (E, J, and O), along with Wallerian degeneration of the left midbrain. No new signal disturbances are present in the parenchyma, and there is no abnormal contrast enhancement.

**Figure 3:**
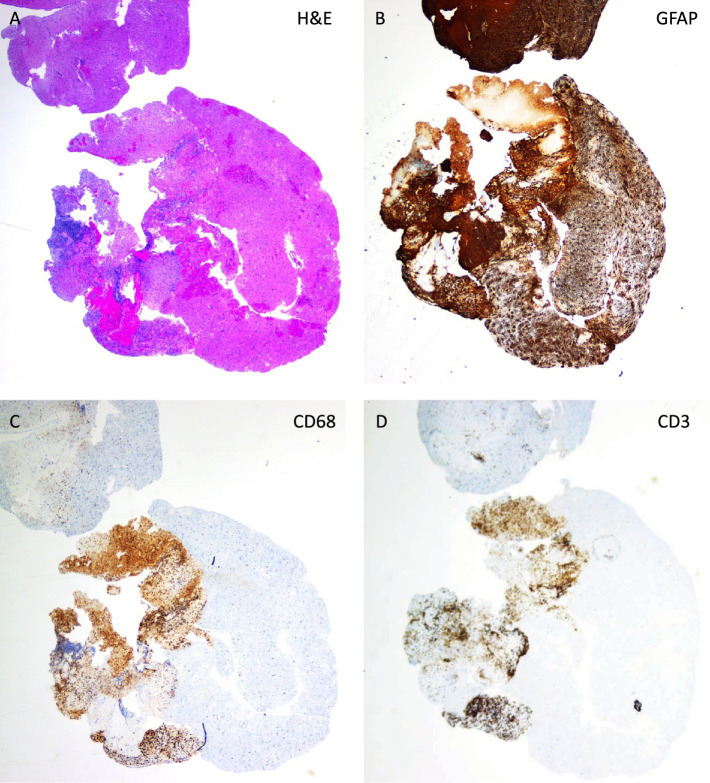
Neuropathological examination of brain biopsy reveals focal inflammation. The hematoxylin and eosin (H&E) staining **(A)** shows increased cell density around the disrupted area of the spheroid-shaped brain tissue section, fitting with necrosis. In the same area the GFAP staining indicates reactive astrogliosis **(B)**, the CD68 staining **(C)** reveals the presence of macrophages and the CD3 staining **(D)** demonstrates T-lymphocytic infiltration.

A brain biopsy from the frontal lesion was performed on day 10. Biopsy material was scarce and contained a necrotic tissue fragment apart from brain tissue with reactive changes with macrophages, lymphocytes and reactive astrocytes apart from extravasated erythrocytes, see Figure 3. The lymphocytes were both located spread out in the tissue as well as focally accumulated. Obvious malignant cells, mitoses or microorganisms were not observed. Multinuclear cells, granulomas and microglial nodules were absent. The biopsy workup included extensive staining panels for neoplastic conditions and evaluations by both a neuropathologist and a haematopathologist who did not identify findings consistent with lymphoma or glioblastoma. In the absence of neutrophil vessel wall infiltration and of hyaline vessel wall necrosis, the tissue changes were not interpreted as representing a vasculitis.

**Figure 4:**
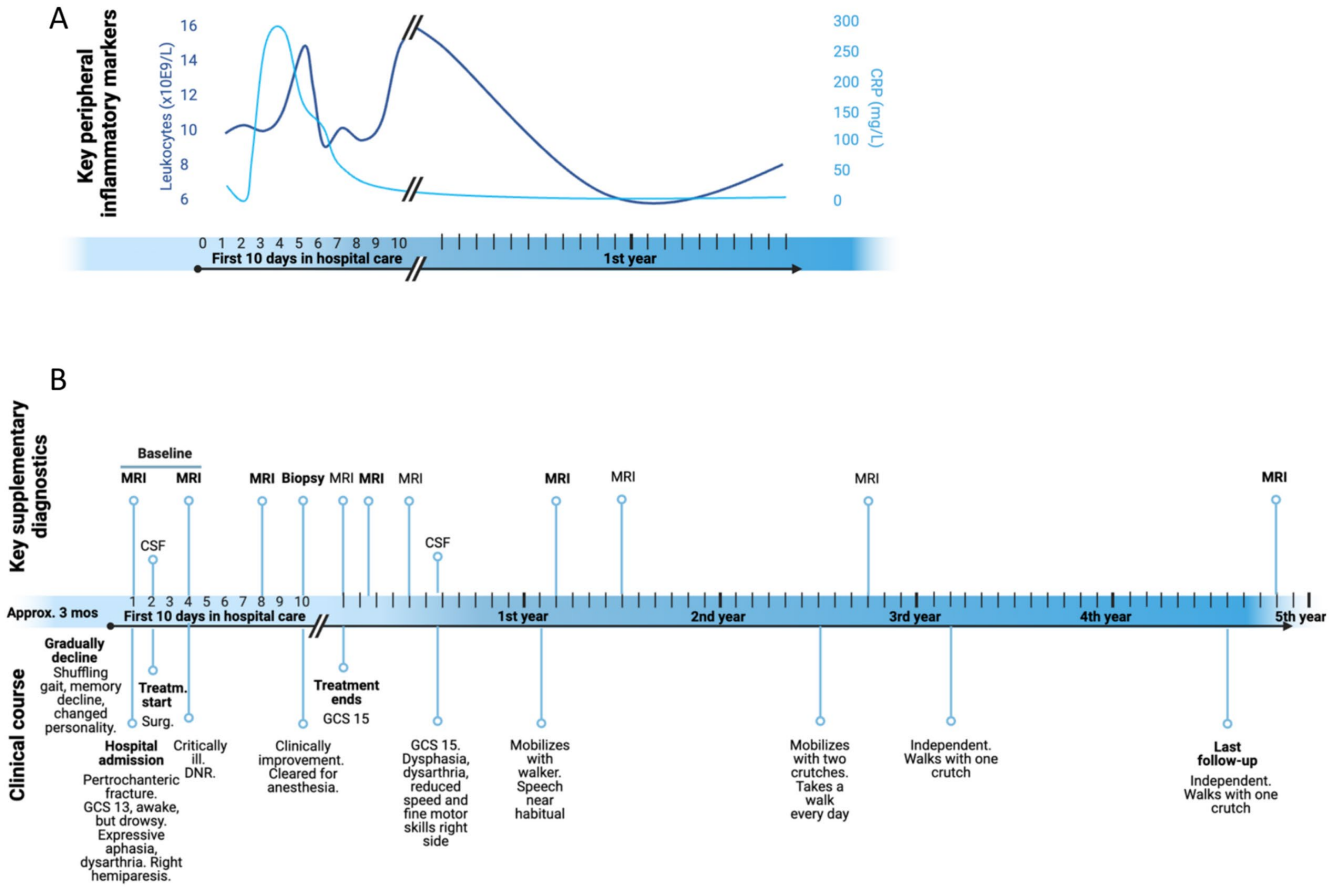
**(A)** Change of key peripheral infection markers over time: leucocyte count (dark blue) and C-reactive protein (light blue). **(B)** Timeline displaying the clinical course of this case and the key supplementary diagnostics that were performed. Generated in BioRender.

Despite a gradual debut of symptoms with hemiparesis leading to fall and fracture, his infection markers [leukocytes and C-reactive protein (CRP)] was normal upon admission, but increased rapidly, accompanied by the development of anemia, see timelines in Figure 4. Cerebrospinal fluid (CSF) tests showed a compromised blood brain barrier (BBB) with elevated protein (2.22 g/L, ref. 0.0–0.45) and albumin (1,447 mg/L, ref. 180–250 mg/L), resulting in an albumin index to plasma above 40 (ref. <10.2), as well as a slight increase in the cell count (8×10^6/L leukocytes in CSF, ref. 0–4), but normal glucose levels. CSF cytology analysis did not reveal any malignant cells.

The possibility of CNS infection was considered. Comprehensive screening for various infectious pathogens was done on plasma, CSF and brain tissue. Borrelia IgM and IgG were negative in both plasma and CSF. John Cunningham-virus (JCV) was not detected in CSF. Treponema pallidum was negative in plasma and therefore not analyzed in CSF. In sections from the brain biopsy, special stains Gram, PAS, Grocotte and Ziehl Neelsen did not reveal bacterial colonies, fungal structures or acid-fast bacilli. Immunohistochemical stains for cytomegalovirus (CMV), SV40-2 (polyomavirus that may exhibit cross-reaction to JCV) and p53, Toxoplasma, Listeria serotypes 1 and 4 were negative. *In situ* hybridization for Epstein Barr Virus (EBV) was negative. Furthermore, the blood culture results showed no growth of aerobic or anaerobic colonies, indicating the absence of bacterial infection in the bloodstream. The patient was HIV and IGRA negative. Furthermore, there were no detectable neuronal antibodies in plasma (neuronal IIF, amphiphysin, CV 2, PNMA2/Ta, Ri, Yo, Hu, Recoverin, Zic4, GAD 65, Tr (DNER), SOX1, titin).

Steroid treatment was initiated directly after surgery for the pertrochanteric fracture on the second day. Post-surgery, the patient was bedridden and barely responsive, in addition to a right-sided hemiparesis. After a week of treatment with oral dexamethasone (4 mg x 4), the patient demonstrated marked improvement. He continued this treatment for 5 weeks and kept improving clinically, enough to be discharged from the hospital midway through the treatment. The BBB and CSF cell count was normalized by the next spinal tap 6 months later, as was his blood leukocytes and CRP levels (see Figure 4A for change of peripheral infection markers over time). Follow-up MRIs revealed a progressive reduction in size, regression of mass effect, hematoma, and edema (see Figure 1).

Upon discharge, still no diagnosis was made. Therefore, prior to the subsequent regular follow-up appointments, interdisciplinary discussions were held involving neurology, neurosurgery, and neuroradiology to determine the appropriate consultation and imaging intervals on an individual basis. The patient showed sustained improvement both clinically and on MRIs without requiring additional medical treatment. During one of these interdisciplinary discussions, conducted almost 3 years after the onset of symptoms, a comprehensive review of all the MRIs was once again conducted. Based on the findings from this review, AHLE was suggested as a possible diagnosis. This prompted a reevaluation of the initial biopsy, which provided further support for AHLE as the correct diagnosis. The combination of the MRI results, clinical presentation and trajectory, biopsy findings, and results from CSF and blood tests ultimately led to the confirmation of AHLE as the final diagnosis in this case.

At the last follow up 5 years after symptom onset, the patient’s dysarthria was minimal, and he no longer experienced communication difficulties. However, cognitive test results indicated slightly impaired word fluency and memory recall, as well as abstraction difficulties. The patient used a walking stick due to an unsteady gait, which was likely partly due to neurological sequelae and partly due to a leg length difference after the pertrochanteric fracture. While the patient still experienced some sequelae, he was able to care for himself at home, engage in daily walks, and socialize with his neighbor.

### Literature review

A search on PubMed was performed on September 11th 2024. The search term “Acute hemorrhagic leukoencephalitis OR “Hurst disease” OR “Hurst syndrome”” was used, and the Advanced Search Builder was used to confirm that the correct MESH term of “leukoencephalitis, acute hemorrhagic” was applied. To be included, the publication had to be in English, include at least one original case, and describe the case clearly as AHLE. The rest of the articles were screened manually, see Supplementary information for flow chart describing selection of publications. This search yielded 152 cases from 1955 to 2024, detailed in a Supplementary Table 1. For each identified case, age, sex, time from debut of illness until initiation of treatment (or alternatively death), and outcome were tabulated, see Supplementary Table 1. Patient outcome was classified using modified Rankin Scale (mRS), where 0 = full recovery, 1–3 = mild to moderate disability, 4–5 = severe disability, and 6 = death.

## Discussion

AHLE is regarded as a post-infectious demyelinating disorder that in most cases lead to a rapid neurological deterioration and high mortality rate (Duggal et al., 2014; Grzonka et al., 2020). AHLE is rare, with a total of 152 cases reported between 1955 and 2023 found through the PubMed library. The rarity of AHLE, coupled with the complexity of its diagnostic workup, contributes to the likelihood of under recognition and underreporting of this life-threatening disease.

Like in this described case, approximately 50% of the patients experience hemiparesis (Alromaihi, 2022). Symptoms tend to rapidly worsen within few days, often culminating in a coma within a week. The median from symptom onset to treatment or death are 2 days across all published cases from 1955 (mean 5.5 days, SD 8.7), see Supplementary Table 1 for literature review. Our case diverges from the typical acute onset, and has the longest time from symptom onset to hospitalization and treatment of all published cases with a gradual onset of symptoms over a three-month period. The longest symptom period described before our patient was 7 weeks (Lee et al., 2005), and only a total of 10 out of the 152 cases report a period of more than 3 weeks (Valentine et al., 1982; Huang et al., 2004; Gillies et al., 1994; Befort et al., 2010; Virmani et al., 2011; Atherton et al., 2016; Bonduelle et al., 2018; Bang et al., 2023). It is important to note, however, that although the patient in the reported case experienced cognitive and motor decline over a three-month period before being admitted to the hospital, his peripheral inflammatory markers were within the normal range upon admission (see Figure 4). There was, however, a notable increase in these markers during the first week of hospitalization. This elevation coincided with not only a clinical deterioration marked by decreased responsiveness, but also the surgical intervention with osteosynthesis and a support plate to stabilize his pertrochanteric fracture.

Our review reveal a slight male predominance in AHLE cases, with 60% affected males compared to 40% females, and a mean age of 36 years among all patients in our literature review. Notably, our patient, at 73 years, represents one of the oldest reported cases with AHLE, with only five previous cases described with a more advanced age. A 76-year-old male with AHLE associated with an EBV infection, achieving a complete recovery following treatment with corticosteroids and acyclovir (Befort et al., 2010), a 75-year-old male with a fulminant course of AHLE resulting in death within 3 days (Chakraborty et al., 2014), a 75-year-old male with lung cancer, leading to death 41 days after onset of neurological symptoms (Mitsuyama et al., 1986), a 75-year-old female that was admitted to a hospital in a moribund and died the same day (Graham et al., 1979), and lastly a 85-year-old male that died of cardiac arrest 11 days after hospitalization. Additionally, ten more cases involving patients above the age of 65 have been reported (Graham et al., 1979; Pinto et al., 2011; Sinzobahamvya et al., 2018; Gobert et al., 2019; Pagano et al., 1999; Barontini et al., 1984; Yamaguchi et al., 2024; Kalafatakis et al., 2024). Given that the demographic aging is a global trend, it becomes paramount to broaden our understanding of AHLE in older patients; as such, our case report provides vital insights that enrich this evolving area of knowledge.

The diagnosis of AHLE poses a significant challenge, given the current absence of formal diagnostic guidelines. Therefore, a comprehensive approach, utilizing neuroimaging, CSF analysis and histopathology is essential for diagnosing AHLE. CSF analysis typically reveal elevated protein levels, leukocytosis, and the presence of red blood cells, however, no universally characteristic pattern has been identified for AHLE (Grzonka et al., 2020). MRIs typically reveal multifocal, variable-sized, poorly defined white matter lesions accompanied by significant edema, space-occupying effects, and petechial hemorrhages (Grzonka et al., 2020). The lesions commonly affect one or both cerebral hemispheres, with a predilection for the parietal and occipital lobes. In certain instances, the lesions extend to involve areas of the brainstem, cerebellum, and deep gray matter (Pinto et al., 2011; Wu et al., 2022). The lesions typically exhibit hyperintensity on T2-weighted and FLAIR images, as well as hypointensity on T1-weighted images, while susceptibility-weighted images (SWI) are particularly useful in highlighting hemorrhages. Diffusion and contrast enhancement characteristics may vary (Varadan et al., 2021). Notably, MRI proves highly effective in differentiating AHLE from ADEM, with lesions identified in AHLE patients typically exhibiting larges size, increased edema, and a more pronounced mass effect (Kuperan et al., 2003). The presence of intraparenchymal hemorrhages also serves as a reliable indicator of AHLE.

Literature describes the histopathological findings in AHLE as features including necrosis, perivascular microhemorrhages arranged in a “ring and ball” pattern, and inflammation containing granulocytes and macrophages. In cases with a prolonged disease course, perivascular demyelination may be observed (Grzonka et al., 2020). Such specific findings as described for AHLE are typically reported from autopsy brain examinations. In contrast, the findings in our case were obtained from three minute brain samples, each measuring no more than 4x4x1 mm. In our case, the tissue fragments contained areas with necrosis, reactive gliosis, and cellular infiltrates containing macrophages and lymphocytes, predominantly CD4+. Neutrophil granulocytes were absent, both in parenchyma, necrotic areas and blood vessels. We observe the description that a predominance of lymphocytes in the lesion is more commonly associated with ADEM, while AHLE is described as more often characterized by a neutrophilic infiltrate (Bang et al., 2023). Relative demyelination was not demonstrable in our minute biopsy tissue (Luxol stains and immunohistochemical stain for neurofilament and myelin basic protein were performed). However, the biopsy location and very small size may in this case not necessarily reflect the most representative and complete features of the radiologically identified lesion. The need for heightened awareness about AHLE is underscored by the fact that many AHLE patients remain undiagnosed until a post-mortem autopsy is performed (Podduturi et al., 2021).

It is important to note that the absence of standardized diagnostic criteria, coupled with the rarity of the condition, frequently leads to the oversight of AHLE as a potential diagnosis, particularly in the early stages. The ambiguous nature of symptoms often directs clinicians towards alternative diagnoses, and even initial MRI scans can be misinterpreted or misleading. Our case vividly illustrates these challenges. Firstly, the unusually slow onset of symptoms caused our patient to go for months without being admitted to any health center. It was only after a fall resulting in a pertrochanteric fracture that his neurological symptoms were investigated. Secondly, despite thorough examination and close follow-up, it still took a considerable amount of time before the correct diagnosis of AHLE was established.

The initial MRI revealed a tumor with hemorrhagic components, low T1 signal, high T2 signal and increased cell density on DWI, and was initially interpreted as a malignant tumor, with lymphoma being the most likely, and glioma and metastasis as possible differential diagnoses. Our case is not the first to illustrate that even though neuroimaging is a helpful tool for diagnosing neurological disorders, it can be misinterpreted. Miller et al. reported a case where, similarly to ours, the MRI scans of an AHLE patient were initially interpreted as a malignant tumor (Miller et al., 1993). Conversely, Schettino et al. described a case of diffuse glioblastoma initially misdiagnosed as AHLE (Schettino et al., 2017). These cases emphasize the critical need for a comprehensive and multifaceted approach when diagnosing neurological disorders. Further investigations in our case, including CSF analysis, brain biopsy, and comprehensive testing for infectious pathogens, ruled out malignancy and infection. Corticosteroid treatment was initiated promptly due to edema, leading to rapid clinical improvement. Increased intracranial pressure is identified as a common cause of death in AHLE patients, and treating this either surgically or medically is believed to positively influence prognosis (Alsaid et al., 2022).

AHLE poses not only a diagnostic challenge, but also a therapeutic one, given the absence of well-established treatment guidelines. No randomized controlled therapeutic trials have been completed, hindered by the rarity of the disease and the rapid clinical deterioration. Consequently, management relies on insights drawn from isolated case studies, as well as general expert knowledge guided by the disease’s proposed pathophysiology. AHLE is primarily recognized as a post-infectious complication that induces autoimmune demyelination (Alromaihi, 2022). The most common precedent is viral upper respiratory viruses, but it has also been linked to other infections, as well as to vaccines (Grzonka et al., 2020). The autoimmune response is postulated to be triggered by molecular mimicry, a form of cross-reaction between the patients’ own tissues and antigens from viral or bacterial pathogens (Alromaihi, 2022), led by the generation of autoantibodies that attack the patient’s brain tissue, causing inflammation, tissue damage, and an increased risk of hemorrhage. Another conceivable autoimmune mechanism thought to be linked with AHLE involves heightened vascular permeability, resulting in perivascular damage and inflammation. This phenomenon may be associated with the formation of circulating immune complexes in response to a pathogen or immunization (Atherton et al., 2016).

The overarching goal of treatment is to reduce the autoimmune inflammation believed to underlie AHLE and prevent secondary neurological damage or death due to intracranial hypertension (Grzonka et al., 2020). First line treatment typically involves immunosuppression, commonly utilizing high-dose corticosteroids and cyclophosphamide. Intravenous immunoglobulins (IVIG) and plasmapheresis (Lann et al., 2010) has also been used. In certain cases, decompressive craniectomy becomes necessary to alleviate intracranial pressure, offering a potential improvement in prognosis (Loesch-Biffar et al., 2021).

There are multiple case reports, including ours, that report improvement after aggressive immunosuppression, although the extent of its efficacy remains unclear (Grzonka et al., 2020). In Grzonka et al.’s systematic review of 43 AHLE-patients, 23 individuals managed to survive (Grzonka et al., 2020): 43% with monotherapy (mainly methylprednisolone), 48% with additional treatments, and 9% with unreported treatment. Of the 20 who did not survive, 30% received monotherapy, 35% had additional treatments, and 35% died before any treatment. This observation highlights that supporting the efficacy of high-dose steroid monotherapy, as demonstrated in our case. The prognosis for AHLE remains generally poor, and in our literature review of all 152 cases in English medical literature, there are a high mortality rate (58%) and death typically occurring only 3 days (median) after the onset of symptoms (Alsaid et al., 2022). Only 14% of patients make a full recovery, in accordance with earlier reports (Grzonka et al., 2020), with a mean modified Rankin outcome scale (mRS) of 4.3 considered a unfavorable outcome (Grzonka et al., 2020; Alromaihi, 2022; Cisse et al., 2011; Khademi and Aelami, 2016), and not changing over the last seven decades. Among those that survive, however, the mean mRS is 1.8, within the range considered a favorable outcome (0–2).

Despite the delayed hospital admission, and hence initiation of treatment, our patient remarkably survived and has shown overall clinical improvement. Our case stands out due to the unprecedented follow-up duration, marking the longest reported in the medical literature for AHLE survivors. Throughout these 5 years, our patient has demonstrated clinical and radiological stability without the need for continued treatment. In our comprehensive analysis of AHLE cases, it is noteworthy that no instances of recurrence have been documented among survivors in the existing medical literature (Alromaihi, 2022), and our case now adds 5 years of observation time to this understanding.

## Conclusion

This report presents a unique case of a 73-year-old AHLE survivor, offering insights into the disease’s presentation and prognosis in older adults. The patient exhibited a prolonged pre-treatment course of a three-months, which is unprecedented in the literature. Despite the high mortality rate of AHLE, our patient’s five-year follow-up shows favorable long-term outcomes. This case underscores the need for heightened awareness and future research to develop guidelines that improve diagnosis and management of this rare condition.

## Data Availability

The original contributions presented in the study are included in the article/Supplementary material, further inquiries can be directed to the corresponding author.
