# Supplementary material for: Acute hemorrhagic leukoencephalitis with gradual symptom onset: Case Report and literature review
Source: Front Neurosci. 2025 Mar 31;19:1557046. doi: 10.3389/fnins.2025.1557046 (PMC11998668; doi:10.3389/fnins.2025.1557046)
Supplement: Supplementary file 1 [file Data_Sheet_1.pdf]

Supplementary – litterature review:

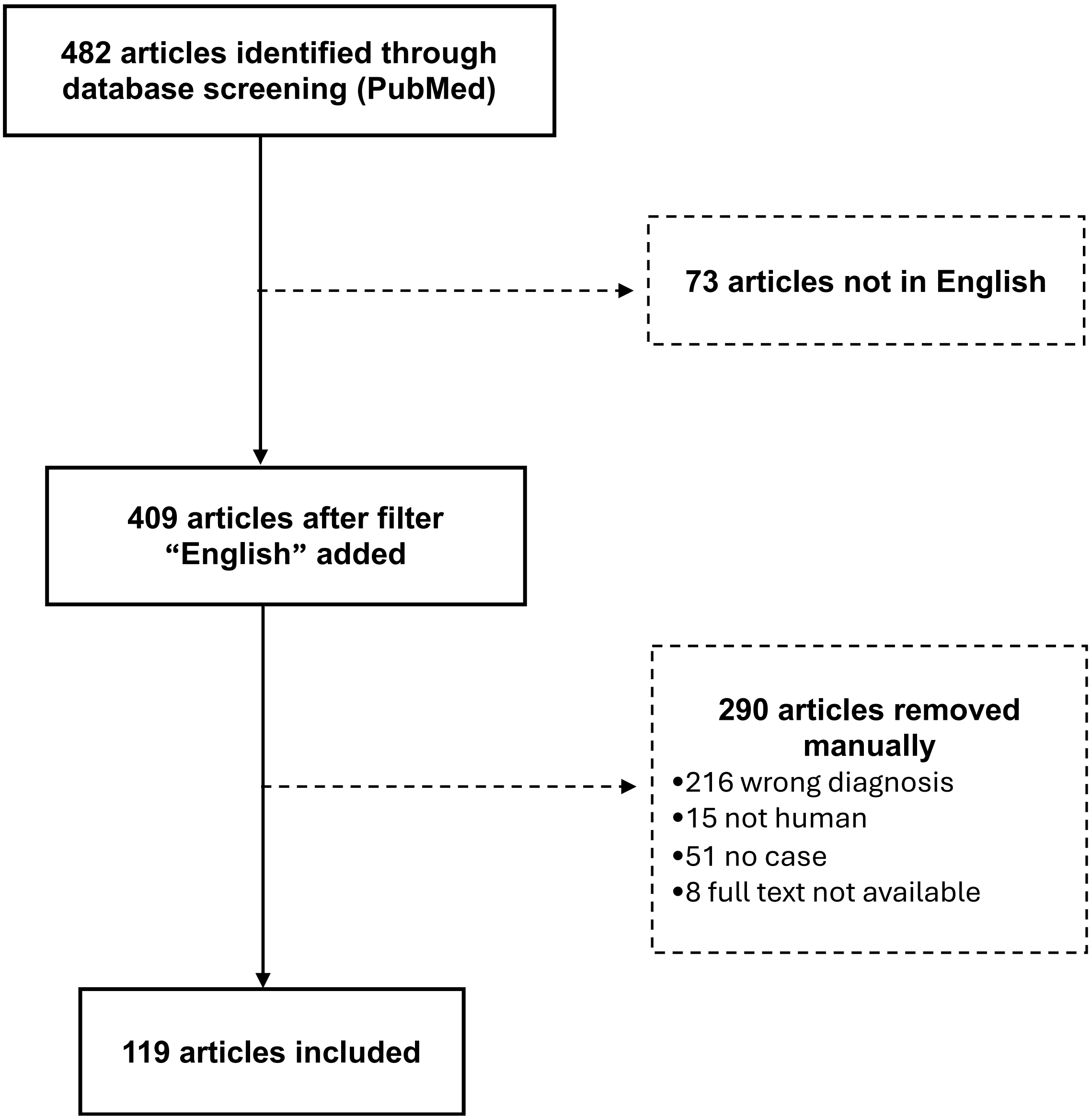

Table with cases form literature review

Year published + source	Age	Sex	Time from debut of symptoms until initiation of treatment or death	Outcome (mRS)
1955 (1)	36	M	2 days	6
1960 (2)	28	M	1 day	6
1960 (2)	32	M	14 days	6
1960 (2)	28	M	<1 day	6
1963 (3)	21	M	2 days	2
1963 (3)	12	F	<1 day	1
1964 (4)	39	M	4 days	2
1973 (5)	56	M	2 days	6
1973 (5)	15	F	5 days	6
1973 (5)	38	F	15 days	6
1975 (6)	2	F	<1 day	6
1975 (6)	15	M	3 days	6
1975 (6)	15	F	3 days	0
1979 (7)	30	M	<1 day	6
1979 (7)	41	M	unclear	6
1979 (7)	53	F	<1 day	6
1979 (7)	71	M	<1 day	6
1979 (7)	72	F	<1 day	6
1979 (7)	75	F	4 days	6
1982 (8)	47	M	<1 day	6
1982 (8)	29	F	6 weeks	0
1982 (8)	23	M	2 days	6
1983 (9)	50	F	1 day	6
1983 (10)	25	M	<1 day	6
1984 (11)	65	F	<1 day	6
1984 (12)	25	M	<1 day	6
1986 (13)	75	M	4 days	6
1988 (14)	22	M	4 weeks	6
1988 (14)	24	M	3 weeks	1
1990 (15)	19	M	<1 day	6
1991 (16)	50	F	<1 day	0
1994 (17)	37	F	<1 day	6
1994 (18)	24	F	3 weeks	6
1997 (19)	6	F	<1 day	0
1997 (20)	34	M	1 day	1
1997 (21)	45	M	3 days	6
1998 (22)	45	M	7 days	6
1999 (23)	71	M	<1 day	6
1999 (23)	44	F	<1 day	6
1999 (23)	70	M	<1 day	6
1999 (24)	53	M	4 days	6

2000 (25)	34	M	<1 day	0
2001 (26)	41	F	3 days	6
2001 (27)	44	F	<7 days	0
2002 (28)	17	M	<1 day	4/5 (paraplegic)
2002 (28)	28	M	3 days	1
2002 (29)	10	F	2 days	1
2002 (30)	5 months	M	<1 day	6
2002 (30)	8 months	M	<1 day	6
2002 (31)	15	F	3 days	6
2003 (32)	19	M	<1 day	6
2004 (33)	57	F	<1 day	4/5 (tetraplegic, global aphasia)
2004 (34)	28	M	3 days	6
2005 (35)	43	M	<1 day	2
2005 (36)	42	F	49 days	1
2005 (37)	3	M	10 days	1
2005 (38)	16	F	<1 day	6
2006 (39)	22	F	2 days	0
2007 (40)	31	M	<1 day	1
2007 (41)	7	F	2 days	1
2009 (42)	30	M	Not reported/unclear	6
2009 (43)	62	M	14 days	2/3
2010 (44)	20	M	8 days	0
2010 (45)	76	M	21 days	0-3 (not specified)
2010 (46)	40	M	«subacute»	5
2010 (47)	21	M	7 days	6
2010 (48)	11	M	8 days	6
2011 (49)	25	M	2 months	1/2 (slight hemiplegia)
2011 (50)	56	F	<1 day	1
2011 (51)	70	M	7 days	6
2011 (52)	37	M	5 days	2
2011 (53)	23	M	7 days	6
2012 (54)	51	M	<7 days	6
2013 (55)	17	F	unknown	1
2013 (55)	10	M	<1 day	3
2013 (56)	3	M	2 days	0
2013 (57)	27	M	2 days	6
2013 (58)	22	M	2 days	6
2014 (59)	75	M	2 days	6
2014 (60)	24	F	unknown	0
2014 (61)	39	M	4 days	6
2015 (62)	48	M	Not reported (found dead)	6
2015 (63)	35	F	Unclear/days	5
2016 (64)	34	F	3-4 weeks	6

2016 (65)	5	M	5 days	6
2016 (66)	13	F	4 days	0
2016 (67)	27	M	<1 day	6
2016 (68)	44	M	3 days	0
2016 (69)	25	F	2 days	4
2016 (70)	33	F	4 days	4
2017 (71)	36	F	<1 day	6
2017 (72)	36	M	2 days	6
2017 (73)	33	F	2 days	1
2018 (74)	25	F	3 weeks	3
2018 (75)	70	M	<1 day	6
2019 (76)	63	M	7 days	5
2019 (77)	42	M	14 days	6
2019 (78)	15	M	1 day	6
2019 (78)	14	M	7 days	0
2019 (79)	8	M	2 days	0-3 (Not speficied)
2019 (80)	68	F	<1 day	4
2020 (81)	85	M	11 days	6
2020 (82)	33	M	5 days	6
2020 (83)	57	M	<1 day	0
2020 (84)	32	F	7 days	6
2020 (85)	51	F	3 days	4
2020 (86)	61	M	7 days	5
2020 (87)	59	M	<1 day	6
2021 (88)	64	M	<1 day	6
2021 (89)	61	M	<1 day	5
2021 (89)	25	F	2 days	4
2021 (89)	55	F	<1 day	6
2021 (90)	56	M	unclear	unclear
2021 (91)	15	F	1 day	4
2021 (92)	59	M	<1 day	6
2021 (92)	47	F	<1 day	5
2021 (93)	6	F	<1 day	0
2021 (94)	43	F	<1 day	2
2021 (94)	56	F	<1 day	1
2021 (95)	14	M	19 days	6
2021 (96)	34	M	3 days	6
2021 (96)	15	M	5 days	6
2021(96)	62	M	5 days	6
2021(96)	28	M	5 days	6
2021 (96)	28	F	Unknown	6
2021 (96)	27	F	4 days	6
2021 (96)	26	F	4 days	5
2021 (96)	17	F	4 days	4
2021 (97)	63	F	unknown	6
2021 (97)	17	M	<1 day	6

2021 (98)	46	M	<1 day	6
2022 (99)	22	M	2 days	5
2022 (100)	"20s"	F	12 days	6
2022 (101)	58	M	20 days	6
2022 (102)	"school age"	F	1 day	0
2022 (103)	64	F	unclear	6
2022 (104)	52	M	14 days	6
2022 (105)	30	M	9 days	0
2022 (106)	8	F	5 days	6
2022 (107)	21	M	3 days	6
2022(108)	33	M	<1 day	6
2023 (109)	9	F	11 days	6
2023 (110)	54	F	3 weeks	0
2023 (111)	29	F	<1 day	0
2023 (112)	"50s"	N/a	1 day	6
2024 (113)	41	M	<1 day	0-4 (not specified)
2024 (114)	30	F	3 days	6
2024 (115)	"20s"	F	<1 day	1-4 (not specified)
2024 (116)	60	F	3 days	6
2024 (117)	68	M	5 days	0
2024 (118)	"50s"	M	<1 day	0
2024 (119)	73	M	7 days	6

Sources

1. Lander H. A case of acute haemorrhagic leucoencephalitis (Hurst) complicating varicella. J Pathol Bacteriol. 1955;70(1):157-65.
2. Vanderfield GK, Tompkins M, Jelhovsky T. Clinicopathological features of acute haemorrhagic leucoencephalitis. Australas Ann Med. 1960;9:29-33.
3. Coxe WS, Luse SA. ACUTE HEMORRHAGIC LEUKOENCEPHALITIS; A CLINICAL AND ELECTRON-MICROSCOPIC REPORT OF 2 PATIENTS TREATED WITH SURGICAL DECOMPRESSION. J Neurosurg. 1963;20:584-96.
4. Martins AN, Kempe LG, Hayes GJ. ACUTE HAEMORRHAGIC LEUCOENCEPHALITIS (HURST) WITH A CONCURRENT PRIMARY HERPES SIMPLEX INFECTION. J Neurol Neurosurg Psychiatry. 1964;27(6):493-501.
5. Gosztonyi G. Acute haemorrhagic leucoencephalitis. Report of three cases. Z Neurol. 1973;204(1):43-66.
6. Byers RK. Acute hemorrhagic leukoencephalitis: report of three cases and review of the literature. Pediatrics. 1975;56(5):727-35.
7. Graham DI, Behan PO, More IA. Brain damage complicating septic shock: acute haemorrhagic leucoencephalitis as a complication of the generalised Shwartzman reaction. J Neurol Neurosurg Psychiatry. 1979;42(1):19-28.
8. Valentine AR, Kendall BE, Harding BN. Computed tomography in acute haemorrhagic leukoencephalitis. Neuroradiology. 1982;22(4):215-9.

9. Fisher RS, Clark AW, Wolinsky JS, Parhad IM, Moses H, Mardiney MR. Postinfectious leukoencephalitis complicating Mycoplasma pneumoniae infection. Arch Neurol. 1983;40(2):109-13.
10. Rothstein TL, Shaw CM. Computerized tomography as a diagnostic aid in acute hemorrhagic leukoencephalitis. Ann Neurol. 1983;13(3):331-3.
11. Barontini F, Di Lollo S, Toscani L. Acute hemorrhagic leukoencephalopathy: clinical and CT diagnosis and histopathological confirmation in an elderly patient. Ital J Neurol Sci. 1984;5(2):215-8.
12. Watson RT, Ballinger WE, Jr., Quisling RG. Acute hemorrhagic leukoencephalitis: diagnosis by computed tomography. Ann Neurol. 1984;15(6):611-2.
13. Mitsuyama Y, Fujimoto S, Kohno T. An autopsied case of acute hemorrhagic leukoencephalitis. Jpn J Psychiatry Neurol. 1986;40(1):105-12.
14. Huang CC, Chu NS, Chen TJ, Shaw CM. Acute haemorrhagic leucoencephalitis with a prolonged clinical course. J Neurol Neurosurg Psychiatry. 1988;51(6):870-4.
15. Pearl PL, Abu-Farsakh H, Starke JR, Dreyer Z, Louis PT, Kirkpatrick JB. Neuropathology of two fatal cases of measles in the 1988-1989 Houston epidemic. Pediatr Neurol. 1990;6(2):126-30.
16. Seales D, Greer M. Acute hemorrhagic leukoencephalitis. A successful recovery. Arch Neurol. 1991;48(10):1086-8.
17. Posey K, Alpert JN, Langford LA, Yeakley JW. Acute hemorrhagic leukoencephalitis: a cause of acute brainstem dysfunction. South Med J. 1994;87(8):851-4.
18. Gillies CG, Grunnet M, Hamilton CW. Tubular inclusions in macrophages in the brain of a patient with acute hemorrhagic leukoencephalitis (Weston-Hurst syndrome). Ultrastruct Pathol. 1994;18(1-2):19-22.
19. Rosman NP, Gottlieb SM, Bernstein CA. Acute hemorrhagic leukoencephalitis: recovery and reversal of magnetic resonance imaging findings in a child. J Child Neurol. 1997;12(7):448-54.
20. Markus R, Brew BJ, Turner J, Pell M. Successful outcome with aggressive treatment of acute haemorrhagic leukoencephalitis. J Neurol Neurosurg Psychiatry. 1997;63(4):551.
21. Chetty KG, Kim RC, Mahutte CK. Acute hemorrhagic leukoencephalitis during treatment for disseminated tuberculosis in a patient with AIDS. Int J Tuberc Lung Dis. 1997;1(6):579-81.
22. Friedman DP. Neuroradiology case of the day. Unilateral acute hemorrhagic leukoencephalitis (AHL) complicated by venous thrombosis. Radiographics. 1998;18(1):246-50.
23. Pagano L, Larocca LM, Vaccario ML, Masullo C, Antinori A, Pierconti F, et al. Acute hemorrhagic leukoencephalitis in patients with acute myeloid leukemia in hematologic complete remission. Haematologica. 1999;84(3):270-4.
24. Case records of the Massachusetts General Hospital. Weekly clinicopathological exercises. Case 1-1999. A 53-year-old man with fever and rapid neurologic deterioration. N Engl J Med. 1999;340(2):127-35.
25. Klein CJ, Wijdicks EF, Earnest Ft. Full recovery after acute hemorrhagic leukoencephalitis (Hurst's disease). J Neurol. 2000;247(12):977-9.
26. Tanser SJ, Walker MB, Hilton DA. Acute haemorrhagic leucoencephalitis complicating sepsis. Anaesth Intensive Care. 2001;29(1):54-7.

27. Meilof JF, Hijdra A, Vermeulen M. Successful recovery after high-dose intravenous methylprednisolone in acute hemorrhagic leukoencephalitis. J Neurol. 2001;248(10):898-9.
28. Pfausler B, Engelhardt K, Kampfl A, Spiss H, Taferner E, Schmutzhard E. Post-infectious central and peripheral nervous system diseases complicating Mycoplasma pneumoniae infection. Report of three cases and review of the literature. Eur J Neurol. 2002;9(1):93-6.
29. Leake JA, Billman GF, Nespeca MP, Duthie SE, Dory CE, Meltzer HS, et al. Pediatric acute hemorrhagic leukoencephalitis: report of a surviving patient and review. Clin Infect Dis. 2002;34(5):699-703.
30. McLeod DR, Snyder F, Bridge P, Pinto A. Acute hemorrhagic leukoencephalitis in male sibs. Am J Med Genet. 2002;107(4):325-9.
31. Takeda H, Isono M, Kobayashi H. Possible acute hemorrhagic leukoencephalitis manifesting as intracerebral hemorrhage on computed tomography--case report. Neurol Med Chir (Tokyo). 2002;42(8):361-3.
32. Kuperan S, Ostrow P, Landi MK, Bakshi R. Acute hemorrhagic leukoencephalitis vs ADEM: FLAIR MRI and neuropathology findings. Neurology. 2003;60(4):721-2.
33. Martins HM, Teixeira AL, Jr., Lana-Peixoto MA. Acute hemorrhagic leukoencephalitis mimicking herpes simplex encephalitis: case report. Arq Neuropsiquiatr. 2004;62(1):139-43.
34. Francisci D, Sensini A, Fratini D, Moretti MV, Luchetta ML, Di Caro A, et al. Acute fatal necrotizing hemorrhagic encephalitis caused by Epstein-Barr virus in a young adult immunocompetent man. J Neurovirol. 2004;10(6):414-7.
35. Gibbs WN, Kreidie MA, Kim RC, Hasso AN. Acute hemorrhagic leukoencephalitis: neuroimaging features and neuropathologic diagnosis. J Comput Assist Tomogr. 2005;29(5):689-93.
36. Lee HY, Chang KH, Kim JH, Na DG, Kwon BJ, Lee KW, et al. Serial MR imaging findings of acute hemorrhagic leukoencephalitis: a case report. AJNR Am J Neuroradiol. 2005;26(8):1996-9.
37. Kabakus N, Gurgoze MK, Yildirim H, Godekmerdan A, Aydin M. Acute hemorrhagic leukoencephalitis manifesting as intracerebral hemorrhage associated with herpes simplex virus type I. J Trop Pediatr. 2005;51(4):245-9.
38. Hofer M, Weber A, Haffner K, Berlis A, Klingel K, Krüger M, et al. Acute hemorrhagic leukoencephalitis (Hurst's disease) linked to Epstein-Barr virus infection. Acta Neuropathol. 2005;109(2):226-30.
39. Alemdar M, Selekler HM, Iseri P, Demirci A, Komsuoglu SS. The importance of EEG and variability of MRI findings in acute hemorrhagic leukoencephalitis. Eur J Neurol. 2006;13(11):e1-3.
40. Ryan LJ, Bowman R, Zantek ND, Sherr G, Maxwell R, Clark HB, et al. Use of therapeutic plasma exchange in the management of acute hemorrhagic leukoencephalitis: a case report and review of the literature. Transfusion. 2007;47(6):981-6.
41. Payne ET, Rutka JT, Ho TK, Halliday WC, Banwell BL. Treatment leading to dramatic recovery in acute hemorrhagic leukoencephalitis. J Child Neurol. 2007;22(1):109-13.
42. Kumar RS, Kuruvilla A. Teaching NeuroImages: Acute hemorrhagic leukoencephalitis after mumps. Neurology. 2009;73(20):e98.

43. Catalan M, Naccarato M, Grandi FC, Capozzoli F, Koscica N, Pizzolato G. Acute hemorrhagic leukoencephalitis with atypical features. Neurol Sci. 2009;30(1):55-7.
44. Takeuchi S, Takasato Y, Masaoka H, Hayakawa T, Otani N, Yoshino Y, et al. Hemorrhagic encephalitis associated with Epstein-Barr virus infection. J Clin Neurosci. 2010;17(1):153-4.
45. Befort P, Gaillard N, Roubille C, Quellec AL. Hemorrhagic leukoencephalitis linked to Epstein-Barr virus in an adult patient. Clin Neurol Neurosurg. 2010;112(9):829-31.
46. Fugate JE, Lam EM, Rabinstein AA, Wijdicks EF. Acute hemorrhagic leukoencephalitis and hypoxic brain injury associated with H1N1 influenza. Arch Neurol. 2010;67(6):756-8.
47. Abou Zeid NE, Burns JD, Wijdicks EF, Giannini C, Keegan BM. Atypical acute hemorrhagic leukoencephalitis (Hurst's disease) presenting with focal hemorrhagic brainstem lesion. Neurocrit Care. 2010;12(1):95-7.
48. Lann MA, Lovell MA, Kleinschmidt-DeMasters BK. Acute Hemorrhagic Leukoencephalitis: A Critical Entity for Forensic Pathologists to Recognize. The American Journal of Forensic Medicine and Pathology. 2010;31(1):7-11.
49. Virmani T, Agarwal A, Klawiter EC. Clinical reasoning: A young adult presents with focal weakness and hemorrhagic brain lesions. Neurology. 2011;76(22):e106-9.
50. Cisse FA, Antoine JC, Pillet S, Jousserand G, Reynaud-Salard M, Camdessanche JP. Acute hemorrhagic leukoencephalopathy associated with influenza A (H1N1) virus. J Neurol. 2011;258(3):513-4.
51. Pinto PS, Taipa R, Moreira B, Correia C, Melo-Pires M. Acute hemorrhagic leukoencephalitis with severe brainstem and spinal cord involvement: MRI features with neuropathological confirmation. J Magn Reson Imaging. 2011;33(4):957-61.
52. Lee NK, Lee BH, Hwang YJ, Kim SY, Lee JY, Joo M. Serial computed tomography and magnetic resonance imaging findings of biphasic acute hemorrhagic leukoencephalitis localized to the brain stem and cerebellum. Jpn J Radiol. 2011;29(3):212-6.
53. Hashim HZ, Ibrahim NM, Wanyahya N, Tan HJ, Zainun KA, Mohd Ali SA, et al. A case of biopsy proven acute demyelinating encephalomyelitis (ADEM) with haemorrhagic leucoencephalitis. Ann Acad Med Singap. 2011;40(4):197-200.
54. Kao HW, Alexandru D, Kim R, Yanni D, Hasso AN. Value of susceptibility-weighted imaging in acute hemorrhagic leukoencephalitis. J Clin Neurosci. 2012;19(12):1740-1.
55. Broderick L, Gandhi C, Mueller JL, Putnam CD, Shayan K, Giclas PC, et al. Mutations of Complement Factor I and Potential Mechanisms of Neuroinflammation in Acute Hemorrhagic Leukoencephalitis. J Clin Immunol. 2013;33(1):162-71.
56. Ichikawa K, Motoi H, Oyama Y, Watanabe Y, Takeshita S. Fulminant form of acute disseminated encephalomyelitis in a child treated with mild hypothermia. Pediatr Int. 2013;55(6):e149-51.
57. Jeganathan N, Fox M, Schneider J, Gurka D, Bleck T. Acute hemorrhagic leukoencephalopathy associated with influenza A (H1N1) virus. Neurocrit Care. 2013;19(2):218-21.
58. Venugopal V, Haider M. First case report of acute hemorrhagic leukoencephalitis following Plasmodium vivax infection. Indian J Med Microbiol. 2013;31(1):79-81.

59. dos Santos MP, Martin J, Woulfe J, Lim SP, Chakraborty S. Autopsy-proven Acute Hemorrhagic Leukoencephalitis in an Elderly Patient. Can J Neurol Sci. 2014;41(1):99-102.
60. Duggal N, Ahmed I, Duggal N. Acute hemorrhagic leukoencephalitis associated with autoimmune myopathy. J Vasc Interv Neurol. 2014;7(4):19-22.
61. Robinson CA, Adiele RC, Tham M, Lucchinetti CF, Popescu BF. Early and widespread injury of astrocytes in the absence of demyelination in acute haemorrhagic leukoencephalitis. Acta Neuropathol Commun. 2014;2:52.
62. Kitulwatte ID, Kim PJ, Pollanen MS. Acute hemorrhagic leukoencephalomyelitis in a man with viral myocarditis. Forensic Sci Med Pathol. 2015;11(3):416-20.
63. Yildiz Ö, Pul R, Raab P, Hartmann C, Skripuletz T, Stangel M. Acute hemorrhagic leukoencephalitis (Weston-Hurst syndrome) in a patient with relapse-remitting multiple sclerosis. J Neuroinflammation. 2015;12:175.
64. Atherton DS, Perez SR, Gundacker ND, Franco R, Han X. Acute disseminated encephalomyelitis presenting as a brainstem encephalitis. Clin Neurol Neurosurg. 2016;143:76-9.
65. Chellathurai A, Ponnusamy S, Periakaruppan A, Gopinathan K, Philson J. Acute Hemorrhagic Leucoencephalitis. Indian J Pediatr. 2016;83(3):276-7.
66. Khademi GR, Aelami MH. Acute Hemorrhagic Leukoencephalitis in Children: A Case Report. Iran J Med Sci. 2016;41(3):245-8.
67. Magun R, Verschoor CP, Bowdish DM, Provias J. Mycoplasma pneumoniae, a trigger for Weston Hurst syndrome. Neurol Neuroimmunol Neuroinflamm. 2016;3(1):e187.
68. Prabhakar AT, Kamanahalli R, Sivadasan A, Joseph E, Viggeswarpu S. Non-fatal acute haemorrhagic leukoencephalitis following snake bite: A case report. Trop Doct. 2016;46(1):57-9.
69. Nabi S, Badshah M, Ahmed S, Nomani AZ. Weston-Hurst syndrome: a rare fulminant form of acute disseminated encephalomyelitis (ADEM). BMJ Case Rep. 2016;2016.
70. Wu CY, Riangwiwat T, Nakamoto BK. Hemorrhagic Longitudinally Extensive Transverse Myelitis. Case Rep Neurol Med. 2016;2016:1596864.
71. George IC, Youn TS, Marcolini EG, Greer DM. Clinical Reasoning: Acute onset facial droop in a 36-year-old pregnant woman. Neurology. 2017;88(24):e240-e4.
72. Peerani R, Berggren M, Herath JC. Sudden Death of a Young Man by Acute Hemorrhagic Leukoencephalitis. Acad Forensic Pathol. 2017;7(3):487-93.
73. Solis WG, Waller SE, Harris AK, Sugo E, Hansen MA, Lechner-Scott J. Favourable Outcome in a 33-Year-Old Female with Acute Haemorrhagic Leukoencephalitis. Case Rep Neurol. 2017;9(1):106-13.
74. Bonduelle T, Stricker J, Minéo JF, Massri A, Guesdon C, Barroso B, et al. Weston-Hurst syndrome with acute hemorrhagic cerebellitis. Clin Neurol Neurosurg. 2018;173:118-9.
75. Sinzobahamvya E, Borrelli S, Rutgers MP, Clause D, Gille M. Acute hemorrhagic leukoencephalitis after seasonal influenza vaccination. Acta Neurol Belg. 2018;118(1):127-9.
76. Mondia MWL, Reyes NGD, Espiritu AI, Pascual V JLR. Acute hemorrhagic leukoencephalitis of Weston Hurst secondary to herpes encephalitis presenting as

status epilepticus: A case report and review of literature. J Clin Neurosci. 2019;67:265-70.
77. Ashraf Z, Todnam N, Morgan J, Rojiani AM. 42-Year-Old Man with Worsening Headache. Brain Pathol. 2019;29(2):305-6.
78. Waak M, Malone S, Sinclair K, Phillips G, Bandodkar S, Wienholt L, et al. Acute Hemorrhagic Leukoencephalopathy: Pathological Features and Cerebrospinal Fluid Cytokine Profiles. Pediatr Neurol. 2019;100:92-6.
79. Panchal A, Perez-Marques F. Intravenous immunoglobulin for acute hemorrhagic leukoencephalitis refractory to plasmapheresis. Clin Case Rep. 2019;7(1):160-3.
80. Gobert F, Ritzenthaler T, André-Obadia N, Dailler F. Do not rely on imaging to predict awakening: The value of neurophysiology in a case of Weston-Hurst syndrome. Clin Neurophysiol. 2019;130(6):960-2.
81. Chu L, Clift F. Anti-CV2/CRMP5 antibody-associated hemorrhagic leukoencephalomyelitis treated with steroids, intravenous immunoglobulin, plasmapheresis, and cyclophosphamide. Mult Scler Relat Disord. 2020;40:101964.
82. Handa R, Nanda S, Prasad A, Anand R, Zutshi D, Dass SK, et al. Covid-19-associated acute haemorrhagic leukoencephalomyelitis. Neurol Sci. 2020;41(11):3023-6.
83. Karapanayiotides T, Geka E, Prassopoulos P, Koutroulou I, Kollaras P, Kiourtzieva E, et al. Concentric demyelination pattern in COVID-19-associated acute haemorrhagic leukoencephalitis: a lurking catastrophe? Brain. 2020;143(12):e100.
84. Sun S, Wang J, Liu M, Liu T, Wang Y. Cerebellar hemorrhage as the primary clinical manifestation of hyperacute disseminated encephalomyelitis: a case report. Acta Neurol Belg. 2020;120(5):1189-92.
85. Arenas RD, Hernandez ES. Pseudotumoral lesion as a manifestation of acute hemorrhagic leukoencephalitis (Weston-Hurst syndrome). Mult Scler Relat Disord. 2020;46:102583.
86. Yong MH, Chan YFZ, Liu J, Sanamandra SK, Kheok SW, Lim KC, et al. A Rare Case of Acute Hemorrhagic Leukoencephalitis in a COVID-19 Patient. J Neurol Sci. 2020;416:117035.
87. Grzonka P, Scholz MC, De Marchis GM, Tisljar K, Rüegg S, Marsch S, et al. Acute Hemorrhagic Leukoencephalitis: A Case and Systematic Review of the Literature. Front Neurol. 2020;11:899.
88. Walker JM, Gilbert AR, Bieniek KF, Richardson TE. COVID-19 Patients With CNS Complications and Neuropathologic Features of Acute Disseminated Encephalomyelitis and Acute Hemorrhagic Leukoencephalopathy. J Neuropathol Exp Neurol. 2021;80(6):628-31.
89. Ancau M, Liesche-Starnecker F, Niederschweiberer J, Krieg SM, Zimmer C, Lingg C, et al. Case Series: Acute Hemorrhagic Encephalomyelitis After SARS-CoV-2 Vaccination. Front Neurol. 2021;12:820049.
90. Haqiqi A, Samuels TL, Lamb FJ, Moharrum T, Myers AE. Acute haemorrhagic leukoencephalitis (Hurst disease) in severe COVID- 19 infection. Brain Behav Immun Health. 2021;12:100208.
91. Kops S, Dunne K, Lowe MC, Jr. All the More Reason to Get a Flu Shot: An Instance of Acute Hemorrhagic Leukoencephalitis in a Patient With Influenza A. Cureus. 2021;13(1):e12885.

92. Alqahtani A, Alaklabi A, Kristjansson S, Alharthi H, Aldhilan S, Alam H. Acute necrotic hemorrhagic leukoencephalitis related to COVID-19: a report of 2 cases. Radiol Case Rep. 2021;16(9):2393-8.
93. Yamamoto N, Kuki I, Nagase S, Inoue T, Nukui M, Okazaki S, et al. Subtotal hemispherotomy for late-onset spasms after anti-myelin oligodendrocyte glycoprotein antibody-positive acute haemorrhagic leukoencephalitis. Epileptic Disord. 2021;23(6):957-60.
94. Loesch-Biffar AM, Junker A, Linn J, Thon N, Heck S, Ottomeyer C, et al. Case Report: Minimal Neurological Deficit of Two Adult Patients With Weston-Hurst Syndrome Due to Early Craniectomy: Case Series and Review of Literature on Craniectomy. Front Neurol. 2021;12:673611.
95. Wellnitz K, Sato Y, Bonthius DJ. Fatal Acute Hemorrhagic Leukoencephalitis Following Immunization Against Human Papillomavirus in a 14-Year-Old Boy. Child Neurol Open. 2021;8:2329048x211016109.
96. Pujari SS, Kulkarni RV, Ojha P, Gursahani R, Nadgir D, Patil S, et al. Acute haemorrhagic leukoencephalitis (AHLE) - our experience and a short review. J Neuroimmunol. 2021;361:577751.
97. Podduturi V, Blessing MM, Joseph DM, Ross JL, Sandberg GD. A Case Series of Acute Hemorrhagic Leukoencephalitis. The American Journal of Forensic Medicine and Pathology. 2021;42(3):263-6.
98. Varadan B, Shankar A, Rajakumar A, Subramanian S, Sathya AC, Hakeem AR, et al. Acute hemorrhagic leukoencephalitis in a COVID-19 patient-a case report with literature review. Neuroradiology. 2021;63(5):653-61.
99. Hutto SK, Rapalino O, Venna N. Spinomedullary Weston Hurst Syndrome After COVID-19 and Influenza Co-Infection: A Case Report. Neurohospitalist. 2022;12(2):337-40.
100. Vasireddy AR, Mehta AM, Seshadri S, Madhyastha SP. Acute haemorrhagic encephalomyelitis following dengue infection. BMJ Case Rep. 2022;15(5).
101. Been Sayeed SKJ, Moniruzzaman M, Mahmud R, Rashid MB, Chandra Das S. Acute Hemorrhagic Leucoencephalitis (AHLE): A Rare CNS Presentation of Mycoplasma pneumoniae. Cureus. 2022;14(10):e30921.
102. Bamnawat H, Khera D, Didel S, Tiwari S. Paediatric acute haemorrhagic leukoencephalitis. BMJ Case Rep. 2022;15(6).
103. Dos Santos DT, Borelli WV, Pinto CB, Wawrzeniak IC, Bianchin MM, Duarte JA. Acute hemorrhagic leukoencephalitis Associated with COVID-19. Arq Neuropsiquiatr. 2022;80(4):448-9.
104. Alsaid HM, Atawneh MAA, Abukhalaf S, Daoud A, Hamadah A, Gharaibeh K. Acute Hemorrhagic Leukoencephalitis - A Rare but Fatal Form of Acute Disseminated Encephalomyelitis - Complicated by Brain Herniation: A Case Report and Literature Review. Am J Case Rep. 2022;23:e935636.
105. Suresh Chandran CJ, Paul MA, Unni M. Unihemispheric Hemorrhagic Leukoencephalitis. Neurol India. 2022;70(1):470-1.
106. Sharma R, Bhagwat C, Suthar R, Goyal K, Angurana SK, Vyas S, et al. Acute Hemorrhagic Leukoencephalitis with COVID-19 Coinfection. Indian J Pediatr. 2022;89(4):420.
107. Lambert N, Lutteri L, Tshibanda L, Bianchi E, Maquet P. Anti-SOX1 antibody-associated acute hemorrhagic leukoencephalitis. J Neurol. 2022;269(6):3359-62.

108. Wu C, Zhang W, Jiao Y, Dong M, Zhou H, Lv Y, et al. Two-year follow-up of a young male with possible acute hemorrhagic leukoencephalitis: A case report. Medicine (Baltimore). 2022;101(48):e32073.
109. Wong AM, Lin JJ, Hsia SH, Lin KL. MRI of fatal course of acute hemorrhagic leukoencephalitis in a child with SARS-CoV-2 omicron BA 2.0 infection. Neuroradiology. 2023;65(7):1179-81.
110. Bang SJ, Kim S, Seok HY. Acute hemorrhagic leukoencephalitis as a new phenotype of myelin oligodendrocyte glycoprotein antibody-associated disease. Neurol Sci. 2023;44(10):3741-3.
111. Garcia-Castellon F, Alonso-Juarez M, Paz-Gómez R, Chen Y, Baizabal-Carvallo JF. Acute hemorrhagic leukoencephalitis in an adult: prominent response with cyclophosphamide. Acta Neurol Belg. 2023;123(4):1585-8.
112. Skarsta L, Nicoletti T, Frick K, Kana V, De Vere-Tyndall A, Weller M, et al. Acute haemorrhagic leucoencephalitis as clinical manifestation of MOG antibody-associated disease. J Neurol Neurosurg Psychiatry. 2023;94(7):583-5.
113. Iltaf Mairajuddin S, Sr., Salim Inshasi JS, Channa RMA, Anwar Siddiqi S, Shaffi Al Madani AAR, Flayyih R. A Rare and Challenging Presentation of Acute Hemorrhagic Leukoencephalitis With Tumefactive Demyelinating Lesions in a 41-Year-Old Male. Cureus. 2024;16(4):e58282.
114. Safan AS, Noorain Z, Atta MA, Thekkoth R, Suliman AM, Fadlelmula A, et al. CASE REPORT: Fulminant acute hemorrhagic Leukoencephalitis (AHLE): A rare and ruinous outcome with cerebral herniation (COVID-19). eNeurologicalSci. 2024;35:100499.
115. Singh L, Acharya SS, Arumugam P, Shetti S, Sharan S. Bilateral Cerebellar Hemorrhages: An Atypical Presentation of Acute Hemorrhagic Encephalomyelitis. Cureus. 2024;16(7):e64857.
116. Tan Z, Lin SZZ, Foong WD, Yong MH. Acute hemorrhagic leukoencephalitis: a case report and systematic review of factors associated with severe disability and death. Neurol Sci. 2024.
117. Yamaguchi Y, Sawaya R, Shichinohe N, Tanei ZI, Yamasaki M, Tomeoka F, et al. Acute Hemorrhagic Leukoencephalitis with Concurrent Retinal Vasculitis in an Elderly Japanese Patient: A Case Report. Intern Med. 2024.
118. Bunting N, Martynoga R, Crayton H, Pandita A. Recovery from acute haemorrhagic leucoencephalitis secondary to COVID-19. BMJ Case Rep. 2024;17(8).
119. Kalafatakis K, Margoni A, Liakou ME, Stenos C, Toulas P, Korkolopoulou P, et al. Acute hemorrhagic leukoencephalitis following the first dose of BNT162b2 vaccine against SARS-CoV-2: A case report. Heliyon. 2024;10(3):e25545.
